# Computer-Aided Detection of Breast Cancer – Have All Bases Been Covered?

**DOI:** 10.4137/bcbcr.s785

**Published:** 2008-05-23

**Authors:** Gautam S. Muralidhar, Tamara Miner Haygood, Tanya W. Stephens, Gary J. Whitman, Alan C. Bovik, Mia K. Markey

**Affiliations:** 1 The University of Texas Department of Biomedical Engineering, Austin, TX 78712; 2 Department of Diagnostic Radiology, The University of Texas M. D. Anderson Cancer Center, Houston, TX 77030; 3 Department of Electrical and Computer Engineering, The University of Texas at Austin, Austin, TX 78712

**Keywords:** computer-aided detection, breast cancer, mammography, radiology

## Abstract

The use of computer-aided detection (CAD) systems in mammography has been the subject of intense research for many years. These systems have been developed with the aim of helping radiologists to detect signs of breast cancer. However, the effectiveness of CAD systems in practice has sparked recent debate. In this commentary, we argue that computer-aided detection will become an increasingly important tool for radiologists in the early detection of breast cancer, but there are some important issues that need to be given greater focus in designing CAD systems if they are to reach their full potential.

Breast cancer is one of the leading causes of death among women in the United States. Screening mammography is currently the most effective tool for early detection of breast cancer. However, interpretation of mammograms can be difficult due to the variable appearance of normal breast tissue. Also, overlapping tissue structures can mask lesions in dense breasts making detection of some lesions difficult. Computer-aided detection systems (CAD) (Castellino, 2005; Sampat, Markey et al. 2005; Nishikawa, 2007) have been developed to assist radiologists in interpreting mammograms. Breast cancer may manifest in various findings—microcalcifications, masses, and architectural distortion. The main driving force of CAD research has been to detect early signs of these manifestations. The first FDA-approved CAD system for mammography was the R2 ImageChecker M1000 CAD system (U. S. Food and Drug Administration 1998) and since then the FDA has approved other CAD systems for mammography such as the iCAD Second Look CAD system (U. S. Food and Drug Administration 2002) and a mammography CAD engine from Eastman Kodak Company (U. S. Food and Drug Administration 2004).

The effectiveness of CAD systems in detecting early cancer has received a lot of attention in recent years (e.g. (Health Imaging News 2008)). Even though CAD systems have been the subject of intense research, a question that one needs to ask is how effective are these systems in daily clinical use. The question is key since, as discussed by Krupinski (Krupinski, 2004), up until about 10 years ago, the use of CAD systems and their evaluation was limited to laboratory settings and most performance evaluations were carried out independent of the radiologist. Most research groups reported the performance of their system acting independently, as opposed to the performance of radiologists assisted by the system in a realistic clinical environment. However, more recently, the focus has shifted to observer performance studies in clinical environments. In one such study conducted by Freer and Ulissey (Freer and Ulissey, 2001), the effect of CAD on the interpretation of screening mammograms was assessed. The CAD system used in this study was the ImageChecker M1000 (version 2.0, R2 Technology) system. Perhaps, the most important component of this study was that it was conducted in a community breast center over a 12-month period and a total of 12,860 screening mammograms were interpreted with the CAD system. Freer and Ulissey concluded that the use of CAD in the interpretation of screening mammograms can help increase the detection of early-stage malignancies without undue effect on the recall rate or positive predictive value for biopsy.

Research groups working on CAD have been enthusiastic about the promise shown by CAD systems in detecting early cancer. However, in a recent controversial study, a multi-center research team led by Dr. Joshua Fenton of the University of California, Davis, found that CAD did not improve diagnostic accuracy. These results were published in the New England Journal of Medicine (NEJM) (Fenton, Taplin et al. 2007). Fenton et al. reported a decrease in diagnostic specificity from 90.2% to 87.2% after incorporating CAD, a decrease in the positive predictive value from 4.1% to 3.2%, and an increase in the biopsy rate by 19.7%. Fenton et al. concluded that there were no significant increases in sensitivity and the cancer detection rate, and that the use of CAD was associated with significantly lower overall accuracy. These conclusions sparked a huge debate on the overall efficacy of CAD systems. The study by Fenton et al. reported results from 43 facilities in three states, but it was only in seven of these facilities that CAD was actually used. All the seven facilities used the ImageChecker CAD system (version unspecified, R2 technology). Furthermore, out of the 429,345 mammograms included in the study, only 7% of the mammograms were read using CAD. Critics attacked the methodology adopted in the study, particularly the low number of mammograms actually read by CAD; they argued that in order to prove statistical significance of the efficacy of CAD, a much larger volume of cases with cancer is essential. Further, in an editorial of the same issue of NEJM, Hall (Hall, 2007) pointed out a possible flaw in the study by Fenton et al.—the time taken for a radiologist to get accustomed to a CAD device was not evaluated in this study and the adjustment to computer-aided detection has been estimated to take weeks to years.

A study published by Mathew Gromet in 2008 (Gromet, 2008) compares CAD to double reading of screening mammograms, offering a strong rebuttal to the study published by Fenton et al. (Fenton, Taplin et al. 2007). The CAD system used in this study was ImageChecker (version 5.3, R2 Technology) and a total of 231,221 mammograms were reviewed between January 2001 and December 2005. Out of these, 112,413 cases (48.6%) were double-read and 118,808 cases (51.4%) were single-read with CAD. This makes the number of mammograms reviewed by CAD four times greater than what was reported by Fenton et al. (Fenton, Taplin et al. 2007). The results of the Gromet study indicate that CAD enhances performance of a single reader, yielding increased sensitivity with only a small increase in the recall rate. In another study, Skanne et al. (Skaane, Kshirsagar et al. 2007) compared the use of a CAD system (Image-Checker, version 8.0, R2 Technology) to independent double reading of paired screen-film and full field digital screening mammograms (FFDM). The use of CAD resulted in an increase in the cancer detection rate for FFDM and for screen-film mammography in breast cancer screening performed with independent double reading. Yang et al. evaluated the performance of the Image Checker M1000 (version 3.1, R2 Technology) CAD system applied to full-field digital mammograms for detection of breast cancers (Yang, Moon et al. 2007). They reported a sensitivity of 95% on the fatty breast group and 98% on the dense breast group and reported an average of 1.80 false positive marks per patient on normal mammograms. In conclusion, the studies of Gromet (Gromet, 2008), Skanne et al. (Skaane, Kshirsagar et al. 2007) and Yang et al. (Yang, Moon et al. 2007), as well as a long history of encouraging laboratory studies, demonstrate the high potential of CAD for detecting early cancer.

Even if a technology is effective in an absolute sense, it may not be cost-effective. The cost effectiveness of CAD with mammography for breast cancer screening is a question of great significance. A recent cost effectiveness study of adding a CAD system to a screening mammography program (Lindfors, McGahan et al. 2006) reported a marginal cost per year of life saved (MCYLS) of $19,058. On the other hand, the MCYLS of screening mammography alone was $16,023, which implies that the MCYLS is 19% greater for CAD added to screening mammography versus screening mammography alone. However, one should not ignore the potential benefits of CAD in early detection of cancers. In the United States, investigators involved in cost-effectiveness studies often suggest that $50,000–$100,000 per life year saved may represent the upper limit of appropriate expenditure. This study goes to show that in spite of an increase in the marginal cost of screening with CAD, it is still well within this acceptable range.

Given the broad scope of evidence to date that supports the potential of CAD, we feel confident that computer-aided detection will become an increasingly important tool for radiologists in the early detection of breast cancer. However, have all bases been covered in their design? We believe that there are still basic research challenges to be addressed.

At a fundamental level, most CAD systems rely on image segmentation followed by feature extraction and classification to characterize the lesion. While such an approach might work for lesions such as microcalcifications, for which there is a large contrast between the lesion and the background, it is less effective for lesions that are more diverse in appearance, such as spiculated lesions and architectural distortion. The efficacy of CAD in detecting different types of breast cancer lesions has been debated. Birdwell et al. (Birdwell, Ikeda et al. 2001) conducted a study in which the characteristics of cancers missed at screening were studied and the ability of CAD (R2 Technology, version 2.0) to detect these cancers was assessed. In all, there were 110 patients with 115 cancers. It was found that on prior mammograms with missed cancers, 35 (30%) of the 115 lesions were calcifications, and 80 of the 115 (70%) were mass lesions, with 32 of the 80 masses (40%) being spiculated or irregular. The most frequently suggested reasons for missed calcifications and masses were dense breast tissue and distracting lesions. CAD correctly identified 30 (86%) of 35 missed calcifications and 58 (73%) of 80 missed masses. While CAD marked most of the cases that were missed by the radiologists and needed a recall, the results also suggest that CAD systems are better at detecting microcalcifications than masses. Clinical studies to evaluate the performance of commercial CAD systems for mass detection have reported sensitivities ranging from 67% to 89% with the false positive rate per image (FPI) ranging from 0.40 to 0.74. Thus, recent studies have emphasized algorithm development for specific lesion types that have traditionally been more difficult to detect. For example, Sampat et al. proposed a model-based framework for the early detection of spiculated lesions on mammography (Sampat, 2008). What makes their approach novel is that they measured the physical properties of spiculated lesions on a number of mammograms and attempted to develop a statistical model from those measurements. The statistical model serves as the basis for determining the parameters of several image-processing algorithms, which are deployed in sequence to aid in the detection of spiculated lesions and architectural distortion.

A challenge facing CAD systems is the high number of false positive markings. Most CAD systems report good sensitivity but at the expense of high false positive rates. More research is required to reduce the false positive rate while maintaining a high degree of sensitivity. The research should take into account the human (radiologist) perception of these false positive markings. There is a good case to argue that CAD systems are already reliable second readers. More often than not, when the computer marks false positives, the radiologists can recognize them as false positives rather easily and quickly, and dismiss them, doing no harm to patient diagnosis. In the future, if the overall accuracy of CAD systems could be improved to approximate that of radiologists, the systems could be used as the first reader to triage cases most in need of review by a radiologist. While this is a sharp departure from the current status quo in mammographic CAD, clinical decision aids of this type are not unheard of in other areas of medicine, a good example of which is the Becton, Dickinson and Company intelligent pap imaging solution, which directs attention to slides that most likely contain abnormalities (Becton, Dickinson and Company, 2008). Slides containing no abnormalities are classified under ‘no further review required’ category and can be archived without any further review by cytotechnologists.

The radiology community is also exploring alternative and adjuvant imaging technologies for breast cancer detection, diagnosis, staging, and treatment monitoring. For instance, ultrasound (Kopans, 1998) is routinely used to further evaluate suspicious abnormalities identified on mammography. Ultrasound is particularly useful for distinguishing between cysts and solid lesions and for examining younger women with dense breasts. Likewise, dynamic contrast enhanced breast magnetic resonance imaging (DCE-MRI) (Schnall, 2001), digital breast tomosynthesis (DBT) (Niklason, Christian et al. 1997), digital breast computed tomography (DBCT) (Boone, Nelson et al; 2001; Yang, Carkaci et al. 2007) and stereomammography (SM) (Getty, Pickett et al. 2001) are rapidly evolving technologies. DBT, DBCT and SM are being pursued with interest as they provide 3-dimensional views of the breast, thereby making up for a key limitation of x-ray mammography: loss of information due to the projection of 3-dimensional structures onto a 2-dimensional image plane. CAD systems must be developed to meet the requirements of these new imaging techniques. One of the challenges facing the development of CAD systems for these new imaging modalities is the lack of a sufficient number of clinical cases for testing from these modalities as they are still in an evolving stage. Ideally, there should be a sufficient number of cases to allow for an unbiased development, training, and testing of CAD schemes. With an enlarged database, techniques can be properly optimized and useful features can be determined.

More focus has to be given to standardized performance evaluation of CAD systems and regular quality assessment of CAD systems. Research studies on CAD systems have to be centered on the needs of the radiologist and performance evaluation must be carried out with the end user in mind. The use of technologies such as eye tracking and visual prompts in the visual search of cancers on mammograms should be given more focus. In a study published by Hatton et al. (Hatton, Wooding et al. 2004), it was reported that visual attention was drawn to the prompted regions identified by the ImageChecker CAD system (version unspecified, R2 technology). Different prompts were compared during screening and it was possible to establish the impact of the prompts on visual search patterns. Solid non-transparent prompts were found to interfere with visual search patterns and distract radiologists while subtle prompts were found to be useful for radiologists interpreting screening mammograms.

Finally, CAD systems need to be integrated into radiology training programs to help radiologists get comfortable with the systems. While evaluating the performance of CAD systems, it is essential to take into account the reader’s training and experience with CAD. CAD aided breast cancer detection training should become an important area of research focus. In one such study, Luo et al. (Luo, Qian et al. 2005) showed that there was a statistically significant difference in each observer’s performance in CAD-aided mammography interpretation before and after radiologists were trained on CAD. Luo et al. concluded that CAD training would influence perception, recognition, and interpretation of early breast cancer and CAD performance studies.

In conclusion, while there is still debate on the magnitude of the impact of CAD systems currently in clinical use, we cannot afford to ignore the potential benefits of CAD as we are facing a crisis in mammography in which women’s access to mammographic screening is being endangered by a shortage of breast imaging specialists. In order for CAD systems to reach their full potential, more emphasis must be given to CAD observer studies, developing novel methods for reducing the number of false positive detections, and integrating CAD into medical education. It is also important to bear in mind that the CAD systems are intended to assist radiologists, but not replace them. The radiologist should be the final judge in determining the final assessment. Any effort that assists radiologists in making more accurate interpretations should be encouraged. Failing to do so would greatly reduce the potential benefit to women’s health, which may be improved in a significant way by the development of effective CAD systems.
